# Octopamine controls starvation resistance, life span and metabolic traits in *Drosophila*

**DOI:** 10.1038/srep35359

**Published:** 2016-10-19

**Authors:** Yong Li, Julia Hoffmann, Yang Li, Flora Stephano, Iris Bruchhaus, Christine Fink, Thomas Roeder

**Affiliations:** 1 Christian-Albrechts University Kiel, Zoology, Molecular Physiology, 24098 Kiel, Germany; 2 Bernhard-Nocht-Institute for Tropical Medicine, Hamburg, Germany; 3 Airway Research Center North (ARCN), Member of the German Center for Lung Research (DZL), Germany

## Abstract

The monoamines octopamine (OA) and tyramine (TA) modulate numerous behaviours and physiological processes in invertebrates. Nevertheless, it is not clear whether these invertebrate counterparts of norepinephrine are important regulators of metabolic and life history traits. We show that flies (*Drosophila melanogaster*) lacking OA are more resistant to starvation, while their overall life span is substantially reduced compared with control flies. In addition, these animals have increased body fat deposits, reduced physical activity and a reduced metabolic resting rate. Increasing the release of OA from internal stores induced the opposite effects. Flies devoid of both OA and TA had normal body fat and metabolic rates, suggesting that OA and TA act antagonistically. Moreover, OA-deficient flies show increased insulin release rates. We inferred that the OA-mediated control of insulin release accounts for a substantial proportion of the alterations observed in these flies. Apparently, OA levels control the balance between thrifty and expenditure metabolic modes. Thus, changes in OA levels in response to external and internal signals orchestrate behaviour and metabolic processes to meet physiological needs. Moreover, chronic deregulation of the corresponding signalling systems in humans may be associated with metabolic disorders, such as obesity or diabetes.

The survival and fitness of animals critically depend on the ability to cope with changing environmental and physiological situations. Well-organised hormonal systems match physiological performance with actual conditions. In vertebrates, this regulatory task is assigned to the HPA (hypothalamic-pituitary-adrenal) axis, including the hormones epinephrine and norepinephrine, which mediate organ reactions to a great variety of stressors. The multifaceted fight-or-flight response is a prime example of a stress reaction in which epinephrine and norepinephrine are critically important^1^. In general, the stress-induced release of these hormones coordinates numerous behavioural, physiological and metabolic reactions. Chronic deregulation of this highly important stress response system is causally associated with numerous metabolic, inflammatory and circulation-associated diseases^2^,^3^. To uncover the molecular framework underlying disease development, a better understanding of the organisation and regulation of the adrenergic system is essential. However, the vertebrate adrenergic system is complex and is characterised by a redundant architecture and multiple feedback loops^4^. The general organisation of adrenergic systems in vertebrates and invertebrates is surprisingly similar, although invertebrate systems are less complex^5^,^6^,^7^. In invertebrates, the monoamines octopamine (OA) and tyramine (TA) take the roles of epinephrine and norepinephrine. Tyrosine is catalysed by tyrosine decarboxylase (Tdc2) to yield TA, which is catalysed by tyramine-β-hydroxylase (TβH) to produce OA. Thus, OA-producing cells always also contain TA^8^. The roles of OA in the control of diverse behaviours, such as learning, memory^9^ or aggression^10^, have been characterised in great detail. Moreover, both signalling compounds are important for the regulation of metabolic traits, similar to mammalian adrenergic systems^11^. Thus, understanding the role of both compounds in the modulation of behaviours and physiological parameters that influence energy intake and/or energy expenditure are of prime importance. Sleep is one behaviour that modulates energy intake/expenditure homeostasis. The duration of sleep as well as the latency to sleep are both under the control of OA and TA^12^, which obviously have a major impact on metabolic activity^13^,^14^. OA has a wake-promoting effect that is presumably mediated by the OAMB receptor located on insulin-producing cells of the *pars intercerebralis*^14^,^15^. Consequently, flies with increased release of both adrenergic compounds, TA and OA, have more fat deposits than matching controls^13^. Physical activities, typical sources of energy expenditure^16^, are controlled to different extents by OA and/or TA. This is particularly apparent for the most energy-demanding type of locomotion, insect flight^17^. One peculiar aspect of insect locomotion, starvation-induced hyperactivity, which is required to find new food sources in times of starvation, critically depends on OA^18^,^19^. In *Drosophila* larvae, OA and TA have opposite effects on movement activity, indicating that the compounds act antagonistically^20^,^21^. These effects are tightly associated with the differential effects of the compounds on the neuromuscular junction; OA enhances muscle performance, whereas TA has a generally inhibitory effect^22^. OA and TA also modulate energy intake. In larvae, proper signalling via OA is required to adjust feeding rates according to physiological needs, thus ensuring an appropriate reaction to hunger, while preventing excessive overfeeding^23^. However, the effects of OA and TA on food intake in adult animals are not unambiguous. The ectopically triggered release of OA and TA enhances food intake^24^, but OA-deficient animals appear to have normal food intake rates^18^. Our knowledge of how OA and/or TA modulate various facets of metabolism is still far from complete. The specific OA receptor OAMB is present on insulin-producing cells in the *pars intercerebralis*, but the connection between OA action and insulin function is still unclear^15^,^25^. Thus, despite the great body of work focused on the roles of OA and TA in invertebrate behaviour, our understanding of the architecture and molecular mechanisms underlying their control of metabolic processes is not complete.

In the current study, we elucidated the roles of OA and TA in the control of major metabolic parameters in the fruit fly *Drosophila melanogaster*. We showed that a lack of OA induces a couch-potato syndrome, characterised by reduced activity, reduced resting metabolic rates, increased body fat and reduced life spans. Increasing OA release had the opposite effects. Moreover, we showed that some of these effects are mediated by the modulation of insulin signalling and that TA acts antagonistically on some metabolic traits. This study provides a deeper understanding of the architecture of this primordial adrenergic system and its relationships with all aspects of energy intake and expenditure. Moreover, it emphasises the vastly underestimated role that chronic deregulation of adrenergic signalling might play in the development of obesity and related disorders.

## Results

**Figure 1:**
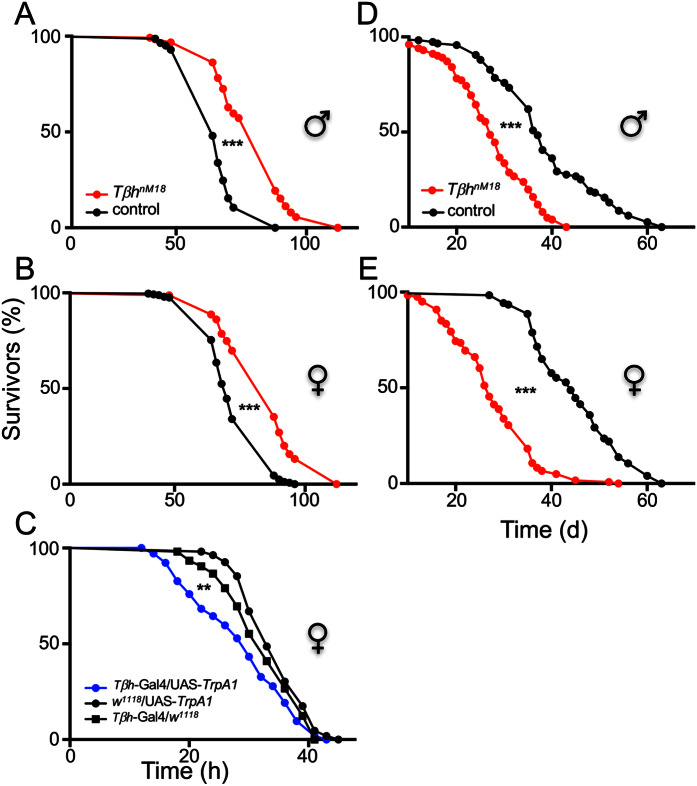
Starvation resistance and life span of animals with altered OA signalling. *Tβh*^*nM18*^ males (**A**) and females (**B**) as well as their matching controls were analysed regarding their starvation resistance. Ectopic activation of OA release by the expression of the temperature-sensitive cation channel TrpA1 (*Tβh*-Gal4 × UAS-*TrpA1*, blue) was compared with that of matching genetic controls (*Tβh*-Gal4 × *w*^*1118*^ and *w*^*1118*^ × UAS-*TrpA1*, black) (**C**). All flies were held at the permissive temperature (30 °C), and at least five independent biological replicates were analysed. Cohorts of male (**D**) and female (**E**) control (black) and *Tβh*^*nM18*^ (red) adult flies were scored for survival. Cohorts of more than 100 animals were used for all survival experiments. Statistical analyses were performed using the log-rank test. *p < 0.05, **p < 0.01 and ***p < 0.001 for all figures.

To elucidate the roles of OA and TA in metabolic control, we used flies defective in the expression of *Tβh (Tβh*^*nM18*^), devoid of OA, and those defective in the expression of *Tdc2 (Tdc2*^*RO54*^), devoid of OA and TA. We also examined matching control flies with the same genetic background. For the majority of our studies, we focussed on flies devoid of OA only, as they allow a more unambiguous interpretation of the results. To evaluate whether the absence of OA has fitness effects, we quantified the starvation resistance of flies lacking OA and those with ectopically triggered OA release. Greater starvation resistance was observed in *Tβh*-deficient animals than matching controls (Fig. 1A,B, log-rank test, p < 0.001). Under conditions of complete starvation, *Tβh*-deficient males and females lived 37% and 26% longer, respectively (Fig. 1A, males: control, 70 h, and *Tβh*-deficient, 90 h; Fig. 1B, females: control, 88 h, and *Tβh*-deficient, 92 h). Consistent with these results, we found that ectopically triggered OA release was associated with a reduced median survival (by approximately 10% relative to controls) in response to complete starvation (controls, 33 h each, and ectopic OA release, 30 h; Fig. 1C, log-rank test, p < 0.01).

The same flies were also analysed with regard to their life span when maintained on normal food. *Tβh*^*nM18*^ flies of both sexes had reduced median as well as maximal life spans compared to those of the matching controls (Fig. 1D,E). Specifically, median life spans decreased by 27% for males (37 d, controls, and 27 d, *Tβh*-deficient; Fig. 1D, log-rank test, p < 0.001) and 39% for females (44 d, controls, and 27 d, *Tβh*-deficient; Fig. 1E, log-rank test, p < 0.001).

### Role of OA in body fat deposition

**Figure 2:**
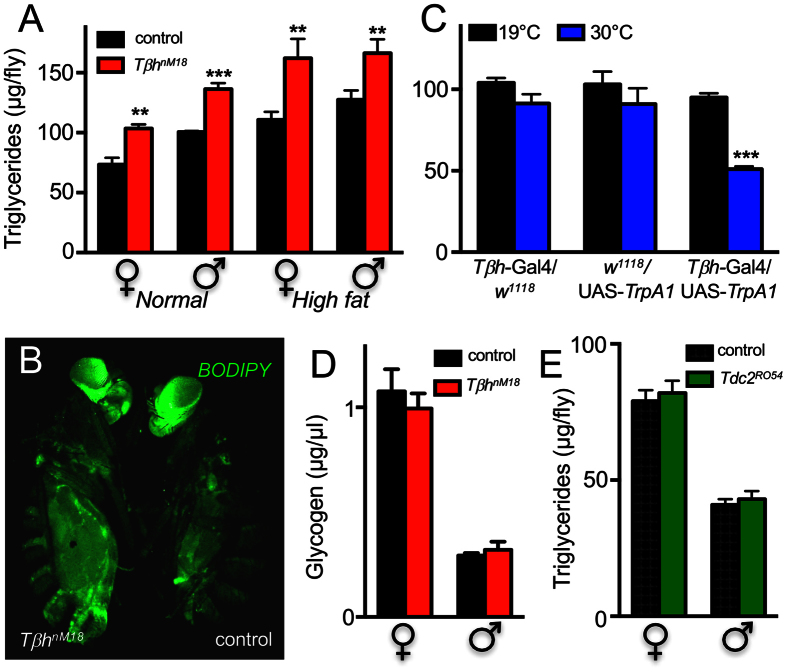
Determination of triglyceride and glycogen levels in flies with altered OA signalling. Triglyceride contents of *Tβh*^*nM18*^ male and female flies challenged with normal (**A**, left) and high-fat diets (**A**, right). BODIPY staining of *Tβh*^*nM18*^ female flies (**B**, left *Tβh*^*nM18*^, right matching control). Triglyceride contents of flies in which the temperature-sensitive TrpA1 channel (UAS-TrpA1) was expressed in OA-producing cells (driven by *Tβh*-Gal4) and the corresponding genetic controls (*Tβh*-Gal4 × *w*^*1118*^ and *w*^*1118*^ × UAS-*TrpA1*) maintained at the restrictive (19 °C, black) or permissive (30 °C, blue) temperature (**C**). Glycogen contents of male and female *Tβh*^*nM18*^ and matching controls are presented (**D**). Triglyceride contents of *Tdc2*^*RO54*^ male and female flies (green) as well as their matching controls (black, **E**). Statistics, see Fig. 1.

We quantified triglyceride levels in *Tβh*-deficient adult males and females to determine whether OA plays an important role in body fat deposition. *Tβh*-deficient flies (*Tβh*^*nM18*^) had significantly higher triglyceride levels than matching control flies (Fig. 2A). We observed a 30% increase in triglyceride levels in both sexes. Moreover, the effect was independent of the nutritional regime; OA-deficient animals had higher triglyceride levels than control flies after feeding on both normal (Fig. 2A, left, p < 0.001) and high-fat (15% fat) media (Fig. 2A, right, p < 0.01). Using a simple BODIPY staining protocol, we visualised the increased fat storage in *Tβh*-deficient flies (Fig. 2B). To specifically increase OA release from OA-producing cells, we directed the expression of the temperature-sensitive TrpA1 channel gene exclusively in these cells (*Tβh*-Gal4 × UAS-*TrpA1*). Triggering OA release for 24 h by temperature shift-induced depolarisation of OA-producing neurons significantly reduced triglyceride levels by approximately 50% relative to the matching genetic controls subjected to an identical temperature protocol (Fig. 2C, p < 0.001). By contrast, we observed no differences between controls and *Tβh*-deficient flies of both sexes with respect to glycogen levels (Fig. 2D). *Tdc2*-deficient animals (males and females), on the other hand, which lacked OA and TA, had body triglyceride levels that were indistinguishable from those of matching controls (Fig. 2E).

**Figure 3:**
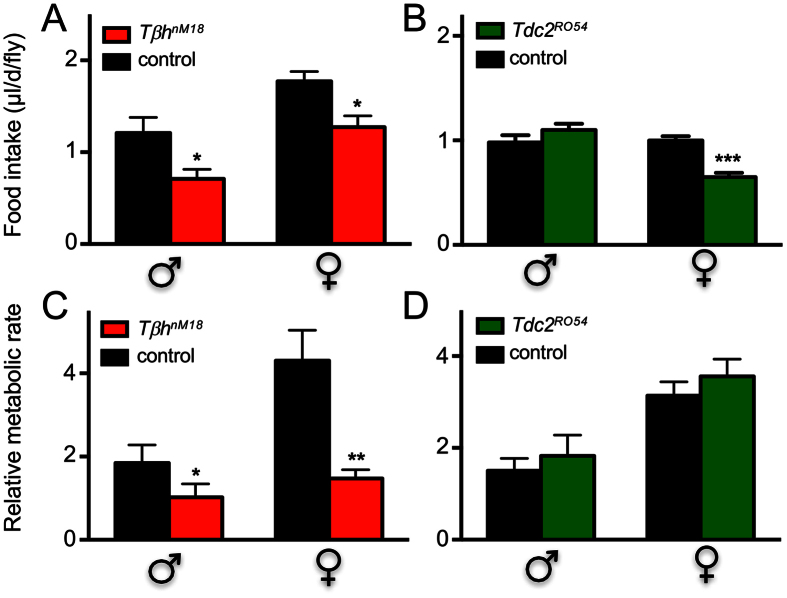
Food intake and metabolic rate of *Tβh*-deficient animals. Food intake of male and female flies was quantified using the CAFE assay (**A**). *Tβh*^*nM18*^ (**A**, red) and control (**A**, black), *Tdc2*^*RO54*^ (**B**, green) and control (**B**, black); the means of four biological replicates ± SEM are shown. Metabolic rates of cohorts of three adult flies were quantified according to Yatsenko *et al.*^27^, using *Tβh*^*nM18*^ and their matching controls (**C**) or *Tdc2*^*RO54*^ and their matching controls (**D**). At least five biological replicates were analysed. Shown are mean values ± SEM. Statistics, see Fig. 1.

Increased energy intake (food consumption) or reduced energy expenditure (e.g., reduced activity or metabolic rate) may explain the high triglyceride levels that we observed in *Tβh*-deficient animals. To assess energy intake, we quantified food intake over 24 h periods using the CAFE assay^26^. We observed approximately 30% lower daily food intake in OA-deficient animals than in controls, for both males and females (Fig. 3A, p < 0.05). Thus, only a strong reduction in energy expenditure can account for the increased body fat in *Tβh*-deficient animals. For *Tdc2*-deficient animals, only females showed a comparable reduction in food intake, while males had almost identical food intake rates to those of controls (Fig. 3B). We analysed the metabolic rates of control and *Tβh*-deficient flies following a recently published protocol to measure CO_2_ production^27^. In this approach, flies are nearly immobilised, facilitating estimates of resting metabolic rates. The metabolic rate was approximately 60% lower in *TβH*-deficient flies than in control flies, and this difference was statistically significant in both males and females (Fig. 3C, p < 0.05 for males and p < 0.01 for females). *Tdc2*-deficient animals, on the other hand, showed nearly identical metabolic rates to those of matching controls (Fig. 3D).

**Figure 4:**
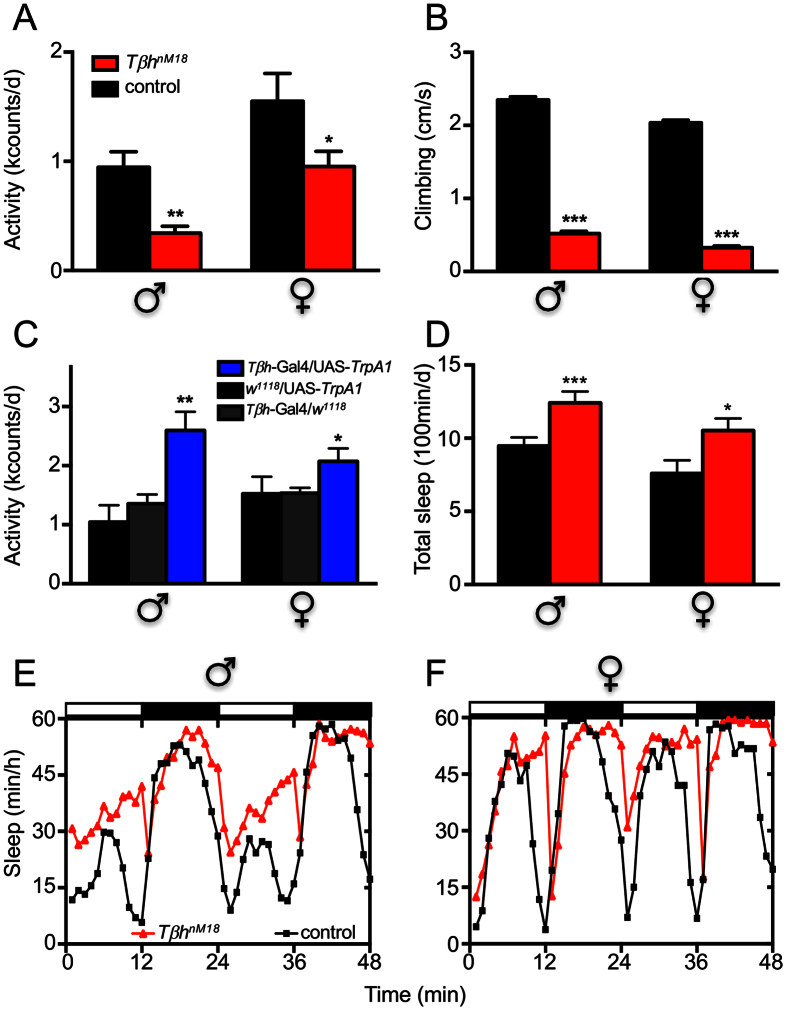
Movement activity and sleep in flies with altered OA signalling. Overall movement activity was analysed using the *Drosophila* activity monitoring system (**A**,**C**–**F**). Twenty-four-hour periods of activity were scored for *Tβh*^*nM18*^ (**A**, red) adults and matching controls (A, black). Climbing performance was tested using a negative geotaxis assay using *Tβh*^*nM18*^ and control flies of both sexes. Animals were scored after fixed time points based on their height on a vertical plane (**B**). Movement activities of animals with ectopically induced release of OA (blue, *Tβh*-Gal4 × UAS-*TrpA1*) were compared with those of both matching genetic controls (grey, *Tβh*-Gal4 × *w*^*1118*^ and *w*^*1118*^ × UAS-*TrpA1*) using the *Drosophila* activity monitoring system. Flies were analysed at 30 °C (**C**). Total sleep time was assessed from the data obtained with the *Drosophila* activity monitor (**D**). For all experiments, mean values of 5–10 independent experiments ± SEM are shown. Sleep activity in representative 48 h intervals is shown for male (**E**) and female flies (**F**). *p < 0.05, **p < 0.01 and ***p < 0.001.

To compare the physical activities *of Tβh*^*nM18*^ flies over longer periods, we used a *Drosophila* activity monitoring system (DAM-system) (Fig. 4A). We detected lower overall movement for *Tβh*^*nM18*^ flies of both sexes than for matching controls (Fig. 4A). We also analysed the ability and/or motivation of flies to walk upwards on a vertical plane in a negative geotaxis assay. In comparison with control animals, the median path length per unit time was reduced by approximately 80% in male and female *Tβh*^*nM18*^ flies (Fig. 4B, p < 0.001). Ectopic activation of OA-release, however, induced increases in overall activity of approximately 30% and 50% in males and females, respectively, compared to control flies (Fig. 4C). Another parameter that influences energy expenditure is sleep. We detected an increased sleep duration (by 25–30%) in *Tβh*-deficient flies of both sexes compared with the corresponding controls (Fig. 4D). Moreover, the latency to sleep was up to 2-fold higher in both male (Fig. 4E) and female flies (Fig. 4F) over a 2 day period.

### Effects of OA on sugar homeostasis and insulin secretion

**Figure 5:**
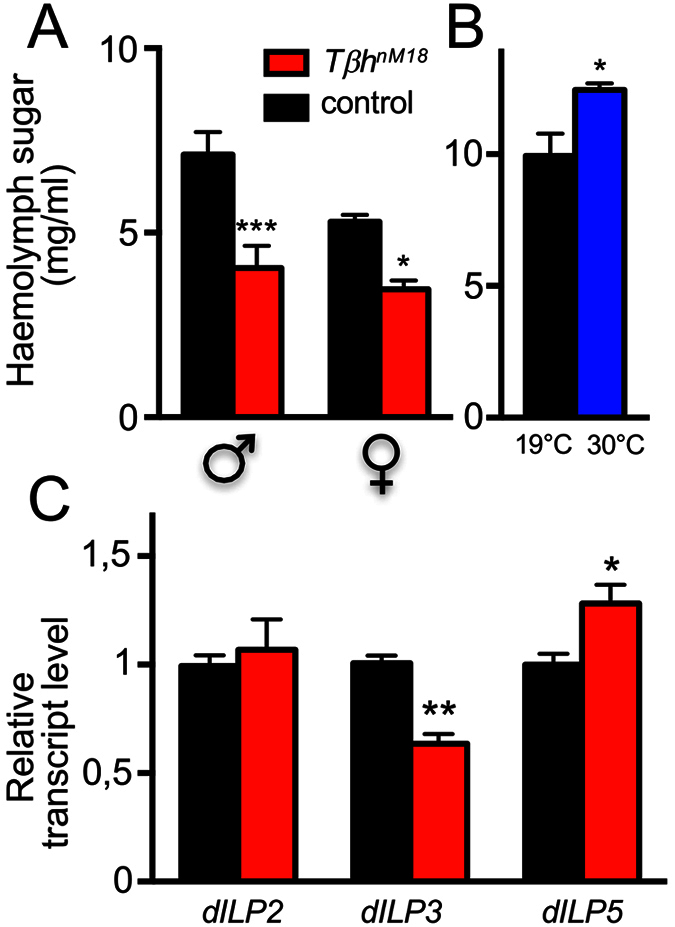
Haemolymph sugar and dILP transcript levels in animals with altered OA levels. Haemolymph sugar levels in *Tβh*^*nM18*^ (red) and matching control (black) flies shown for males and females (**A**). Effect of ectopic release of OA on sugar levels by the application of the permissive temperature (30 °C) to *Tβh*-Gal4 × UAS-*TrpA1* animals compared with genetically identical animals held at the restrictive temperature (19 °C; **B**). Relative transcript levels of dILP2, dILP3 and dILP5 in control and *Tβh*^*nM18*^ flies (**C**), mean values ± SEM of four biological replicates.

The observed metabolic changes may be explained by the OA-dependent deregulation of insulin secretion. To test this hypothesis, we measured haemolymph sugar concentrations and observed statistically significantly lower haemolymph sugar concentrations (both trehalose and glucose) in *Tβh*^*nM18*^ flies (males and females) than in matching controls (Fig. 5A, p < 0.001 for males and p < 0.05 for females). By contrast, increased OA release via activation of the TrpA1 channel in *Tβh*-expressing cells at 30 °C led to an increase in haemolymph sugar concentrations (Fig. 5B, p < 0.05). To assess whether the production or the release of insulin is altered in OA-deficient flies, we quantified transcript levels of the three major insulin-like peptides present in the brain, *dILP2*, *dILP3* and *dILP5*. While the levels of *dILP2* transcripts, the most important insulin-like peptide, were almost identical in controls and *Tβh*-deficient animals, *dILP3* transcript levels were significantly lower, while *dILP5* transcript levels were higher, in *Tβh*-deficient animals than in controls (Fig. 5C, p < 0.01 for both *dILP3* and p < 0.05 for *dILP5*).

**Figure 6:**
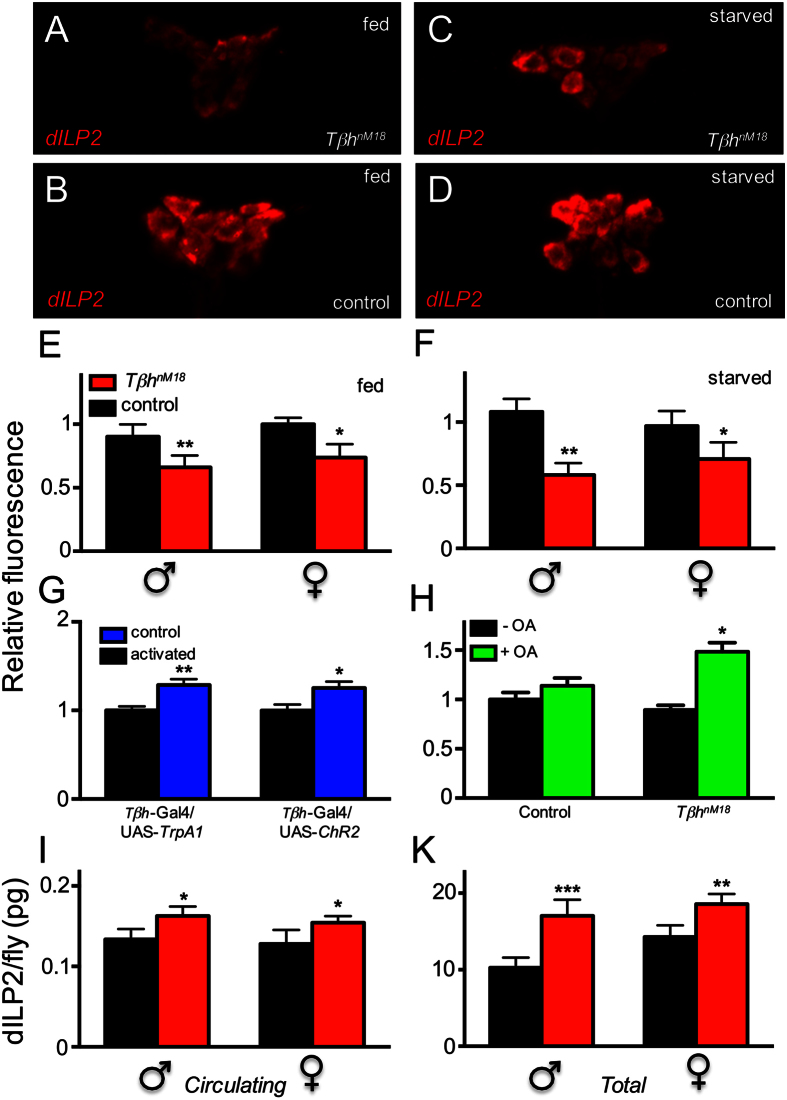
Insulin release in flies with altered OA signalling. Immunohistochemical analysis of dILP2 levels in the ILP-producing cells of the *pars intercerebralis* of *Tβh*^*nM18*^ males and females as well as their matching controls under normal food conditions (**A**,**B**,**E**) as well as following a short period (24 h) of starvation (**C**,**D**,**F**). Representative immunohistochemical pictures (**A**–**D**) and a quantitative evaluation (**E,F**) are shown. Ectopic activation of OA release (*Tβh*-Gal4) by heat in TrpA1 (UAS-*TrpA1*)-expressing cells (**G**, left) or by blue light in channelrhodopsin-2 (UAS-*ChR2*)-expressing cells (**G**, right). Effect of exogenously applied OA on relative dILP2 concentrations in ILP-producing cells of control as well as of *Tβh*^*nM18*^ flies (**H**). Direct measurement of insulin released into the haemolymph (**I**) and total insulin levels (**K**) using flies carrying HA- and FLAG-tagged-dILP2. Mean values of at least five different experiments ± SEM are shown. *p < 0.05, **p < 0.01 and ***p < 0.001.

We assessed dILP2 release from insulin-producing cells using both indirect and direct approaches (Fig. 6). Utilising an indirect approach, we used a specific antibody against dILP2 to quantify the signals in the *pars intercerebralis*, the region containing insulin-producing cells. High signal strengths indicate low release rates, whereas low signal strengths are associated with high release rates^28^. We analysed *Tβh*-deficient and matching control animals of both sexes under fed and starved conditions (Fig. 6A–F). Examples of immunohistochemical images are shown in Fig. 6A–D. We detected a statistically significantly lower dILP2 signal in insulin-producing cells of *Tβh*-deficient animals than in cells of controls in both sexes, indicating a significant increase in insulin release under both fed (Fig. 6A,B,E) and starved conditions (Fig. 6C,D,F). Under control conditions, the dILP2 signal was reduced by 26.8% in males (p < 0.01) and 26.1% in females (p < 0.05, Fig. 6E). In response to starvation, the reduction was 46.2% for males (p < 0.01) and 27.9% for females (p < 0.05, (Fig. 6F). Triggering OA release from *Tβh*-positive cells by shifting the temperature of *Tβh*-Gal4 × UAS-*TrpA1* F1 animals induced the opposite effects, as seen in *Tβh*-deficient animals, i.e., we detected high anti-dILP2 immunoreactivity, indicative of reduced insulin release (Fig. 6G, left, p < 0.01). We observed an almost identical effect when we induced depolarisation of *Tβh*-expressing cells by channelrhodopsin-2, which is blue light-sensitive. *Tβh*-Gal4 × UAS-*ChR2* flies were exposed to blue-light radiation, resulting in the depolarisation and release of OA (Fig. 6H, right, p < 0.05). The insulin levels in *Tβh*-deficient animals could be enhanced substantially by the application of OA, implying that increased OA levels decreased insulin release (Fig. 6H, p < 0.05). To confirm the results, we directly measured the total dILP2 levels as well as the levels released into the haemolymph using an approach that was introduced recently^29^. The levels of circulating insulin were significantly higher in *Tβh*-deficient males and females than in controls, which supports the results obtained using indirect measurements described above (Fig. 6I). Moreover, *Tβh*-deficient flies of both sexes had higher total dILP2-levels than matching controls (Fig. 6K).

## Discussion

OA and TA, the invertebrate equivalents of norepinephrine and epinephrine, are major regulators of metabolism, behaviour and fitness in *Drosophila*. Flies devoid of OA (*Tβh*^*nM18*^) develop a typical couch-potato syndrome, characterised by increased body fat combined with low physical activity. Moreover, these flies show a substantially altered fitness with a reduced overall life span and increased starvation resistance. In human patients, chronic administration of *β*-blockers, which specifically block signalling through *β*-adrenergic receptors, induces comparable phenotypes, including reduced physical activity, a reduced metabolic rate and an increase in body fat^11^. Very similar results can also be observed in mice that are deficient in all three *β*-adrenergic receptors; most importantly, these animals develop severe obesity^30^. We showed that, in *Drosophila*, ectopically triggered OA release consistently led to lean and active animals, similar to rats treated with adrenergic agonists^31^. Flies devoid of both OA and TA did not show this couch-potato phenotype, indicating that OA and TA act antagonistically on major metabolic traits, and particularly on body fat deposition. While OA release reduced body fat, we inferred that TA release has the opposite effect, rescuing the fat deposition phenotype clearly seen in *Tβh*-deficient *Drosophila*. This antagonistic behaviour of OA and TA might explain the discrepancies between our results and those of Erion and colleagues^13^, who reported increased body fat in flies with ectopically triggered release of both OA and TA.

To increase body fat, either energy intake has to increase or energy expenditure has to decrease^16^. In flies depleted of OA, increased body fat appears to occur mainly by the latter mechanism because energy or food intake is reduced. In contrast to our results, Yang and colleagues found no change in food intake in *Tβh*-deficient animals^18^; the difference between studies might be explained by differences in experimental setups. Nevertheless, the major conclusion that the increase in body fat is not the result of increased food intake is supported by both studies. Physical activity and basal metabolic rate are the two major components that contribute to energy expenditure. Both components are controlled by OA, which is obviously a phylogenetically highly conserved feature of adrenergic signalling systems, as adrenaline/noradrenaline have almost identical effects in mammals^32^. Increased physical activity mediated by OA release has been studied in the context of starvation-induced hyperactivity, where OA acts as a stress hormone in periods of starvation and triggers this energy-consuming behaviour^18^,^19^. These effects of OA may, at least in part, be explained by an OA-mediated increase in muscle contraction and output. Interestingly, TA has exactly the opposite effect, which can explain the antagonistic functions of these compounds in different paradigms^22^. In addition to physical activity, the resting metabolic rate is the major factor that contributes to energy expenditure. We showed, for the first time, that the level of OA release determines the resting metabolic rate in insects. Again, TA and OA appear to have antagonistic effects. The tight dependence of the metabolic rate and OA level is very similar to the situation in vertebrates, where epinephrine release or administration substantially increases the metabolic rate, while blocking adrenergic signalling has the opposite effect^11^,^33^.

OA and TA, exactly as epinephrine and norepinephrine, act as neurohormones and are released in response to various stressors to improve physical performance as part of the fight-or-flight response. Applying stressors such as chronic physical agitation to crickets leads to a long-lasting increase in circulating OA and, as a consequence, to a decrease in fat stores^34^. This complex mechanism evolved from very simple reactions to primordial stressors. Starvation is a very simple stressor that increases OA-mediated signalling in the nematode *Caenorhabditis elegans*^35^. Recently, this link between starvation and the more complex regulatory effects of OA was also shown in fruit flies^18^. Taken together, OA appears to be a central mediator of stress responses not only in invertebrates, but also in vertebrates, where epinephrine and norepinephrine take the role of OA as a major stress-induced hormone^36^.

We observed interesting fitness effects in animals with deregulated OA signalling. A lack of OA decreased the life span of adult flies. These differences in life span may be correlated with changes in insulin signalling, a well-known regulator of this trait. Generally, reduced insulin signalling is associated with a longer life span, while increased insulin signalling has the opposite effect^37^. As indicated above, flies devoid of OA had lower haemolymph glucose concentrations and higher insulin release rates, which was shown here the first time in insects. These findings are consistent with previous observations in many other model organisms that increased insulin signalling is associated with reduced life span. Similarly, triple ß1-3-knockout mice also have substantially reduced blood glucose levels^38^. A similar relationship is predicted for flies with high rates of OA release. This connection between OA and insulin secretion was bolstered by the finding that receptors for OA are present in the *pars intercerebralis*, the cells in the fly brain that produce, store and release insulin^39^. Moreover, the OAMB receptor in insulin-producing cells of the *Drosophila* brain may be a molecular substrate required to execute the effects of OA on insulin secretion, although the deregulation of OAMB expression in insulin-producing cells did not directly affect haemolymph glucose according to Luo and colleagues^15^.

Taken together, these results imply that high circulating OA levels are beneficial for animals as they allow for a healthy, active and long life. However, these advantages are linked with an important fitness cost, namely, reduced starvation resistance. Flies devoid of OA have more fat and significantly higher starvation resistance than flies with OA, and these phenotypes might enable flies to survive periods of hunger. For example, these flies can survive adverse weather, while those with a higher level of OA might not be able to survive such periods of starvation. If these results can be applied to humans, individuals with different basal levels of norepinephrine release would tend to have more body fat owing to reduced movement and metabolic rates. During human evolution, this may have been advantageous for survival during periods of starvation. However, individuals with high OA release may be fitter in modern societies, where periods of starvation are usually never experienced.

## Conclusion

We conclude that octopamine signalling regulates the switch between two metabolic states, a thrifty one characterised by reduced energy expenditure and increased body fat deposition and an expenditure mode with the opposite phenotypes. Moreover, flies with low levels of octopamine have reduced life spans, but substantially increased starvation resistance. At least some of the phenotypes induced by octopaminergic signalling are presumably mediated by the direct regulation of insulin signalling.

## Methods

### Fly stocks and husbandry

*Drosophila* stocks used in this study were obtained from the following sources: the tyramine-*β*-hydroxylase (TβH) null mutant line^8^, *Tβh*^*nM18*^ (supplied by Henrike Scholz, Cologne, Germany), was described previously^8^. This line was backcrossed several times with *w*^*1118*^, the control line for all corresponding experiments. *Tdc2*^*RO54*^ as well as flies with the matching genetic background were provided by Jay Hirsh (University of Virginia, USA) and have been described previously^40^. The following transgenic lines were from the Bloomington *Drosophila* Stock Center: *Tβh*-GAL4 (#45904), *Tdc2*-GAL4 (#9313), UAS-*dTrpA1* (#26263) and UAS-*ChR2* (#9681). Unless otherwise stated, the flies were raised on standard medium at 25 °C with 50–60% relative humidity under a 12:12 h light/dark cycle as described earlier^41^. All assays utilised virgin females at a density of 20–25 adults per vial.

### Life span analysis

Life span assays were essentially performed as described earlier^42^. In brief, adult female flies were separated into new vials within 8 h after eclosion. Flies were kept under highly standardised conditions (25 °C, 12 h/12 h light/dark, 65% humidity). For each genotype, four to five replicate cages were combined to analyse survival. Flies were transferred to fresh medium every other day, and the number of dead flies was recorded.

### Stress resistance assays

For the starvation resistance assay, 4–5-day-old flies were used. Approximately 25 flies of each sex were placed in vials containing 1% agar, all vials were incubated under constant conditions (25 °C, 12 h/12 h light/dark, 65% humidity) and dead flies were counted every 2 h until all flies were dead. Experiments involving dTrpA1 activation were assayed at 30 °C.

### Locomotor activity test

The *Drosophila* Activity Monitoring System (DAM-System, TriKinetics Inc., Waltham, MA, USA) was used to evaluate locomotor activity. Newly hatched flies were kept on fresh medium for 3 days before measurements were obtained. The total activity levels of ten flies in one vial (three vials per genotype) were measured under constant conditions (25 °C, 12 h/12 h light/dark, 65% humidity) in a horizontal direction for 3 days, and the data were collected and recorded at 5 min intervals. The total locomotor activity was identified as the sum of the movement during the testing period.

For the negative geotaxis assay, 20 flies of each genotype were transferred into a 20 cm-tall glass tube without CO_2_ anaesthesia. The tubes were tapped to move flies to the bottom, and the climbing height of flies was captured photographically 5 s afterwards. Four to five replicates per genotype were tested under identical conditions.

### Body fat and glycogen quantification

Total body triacylglycerols (TAGs) in flies were measured using the coupled colorimetric assay as described previously^42^,^43^. Glyceryl trioleate served as the TAG standard. Five females (or eight males) per group were weighed and homogenised in 1 ml of 0.05% Tween-20 using a Bead Ruptor 24 (BioLab Products, Bebensee, Germany). Homogenates were heat-inactivated at 70 °C for 5 min, centrifuged and incubated with triglyceride solution (Fisher Scientific, Waltham, MA, USA) at 37 °C for 30 min with shaking. Absorbance was read at 562 nm, and the quantity was estimated using the standard curve. Each measurement was performed with at least three biological replicates.

Glycogen was determined using the Glycogen Assay Kit (Sigma, Deisenhofen, Germany), according to the manufacturer’s instructions. Homogenisation was performed with five animals per sample using the Bead Ruptor 24 (BioLab Products). Six biological replicates were analysed for each condition, and mean values ±SEM are given.

### Metabolic rate determination

The relative quantification of metabolic rates was performed according to the methods of Yatsenko and colleagues^27^. In brief, the metabolic rate of three adult flies per vial was measured for 2 h using respirometry. Data were calculated based on the volume of CO_2_ production during the test and are presented as μl per h per fly. Data obtained from five independent biological replicates were combined.

### Glucose and trehalose measurement

Glucose and trehalose were measured using the Glucose (HK) Assay Kit (Sigma), with minor modifications. Haemolymph samples were pooled from 15–20 flies of each genotype as described previously^44^. The sample was diluted 1:10 in deionised water and added to 50 μl of glucose standard solution, followed by incubation for 15 min at room temperature. Total glucose was recorded by measuring absorbance at 340 nm. For trehalose measurements, 0.25 μl of porcine kidney trehalase (Sigma; 1:200) was added to convert trehalose to glucose, and samples were incubated at 37 °C overnight. Absorbance was measured again, and the amount of trehalose was calculated.

### CAFE assay

The Capillary Feeder (CAFE) assay was performed as previously described^26^, with minor modifications. In brief, a 5 μl glass capillary was filled with 10% glucose as a liquid food source. For each assay, two 7-day-old flies were transferred to the internal tube and allowed to recover for 1 day prior to obtaining measurements. The experiment was carried out under constant conditions (25 °C, 12 h/12 h light/dark, 65% humidity). The descent of the top meniscus of liquid was monitored for 24 h and the volume consumed is expressed in units of μl per fly.

### Immunohistochemistry and direct dILP2 measurements

Adult brains were dissected in Haemolymph-like saline (HL-3) buffer, fixed in 4% paraformaldehyde for 30 min at room temperature, rinsed three times for 5 min each in PBT (0.3% Triton X-100) and blocked in blocking-buffer (1 ×  HL-3) for 30 min at room temperature. Rabbit anti-dILP2 (a gift from Eric Rulifson, UCSF, USA)^45^ diluted 1:200 was added and incubated overnight at 4 °C. After washing three times, diluted Alexa594-labelled donkey anti-rabbit IgG (Jackson ImmunoLabs, Suffolk, UK) was added and incubated for 3 h at room temperature, followed by three additional washing steps. Finally, the tissue was mounted on a slide and images were obtained using a fluorescent microscope equipped with an apotome (Carl Zeiss Image AxioVision, Göttingen, Germany).

To measure fluorescence intensity, optical tissue sections were used. To facilitate the quantification of fluorescence intensities, series of sections (2 μm-thick) covering the entire dILP2-positive region in the *pars intercerebralis* were gathered under identical conditions; exposure times and all other relevant settings were identical for each section. Fluorescence intensity was quantified using ImageJ (National Institutes of Health, Bethesda, MD, USA).

To quantify circulating as well as total dILP2 levels, the method described in Park *et al.*^29^ was used. Flies carrying the FLAG- and HA-tagged version of dILP2 on the second chromosome were crossed with *Tβh*^*nM18*^ as well as their matching controls to generate *Tβh*-deficient males and females with only one *Tβh* allele as well as one gd2HF(attP2) allele to allow the quantification of circulating and total amounts of dILP. Haemolymph isolation as well as ELISA were performed exactly as described in Park *et al.*^29^.

### Quantitative RT-PCR

Total RNA was extracted from the brains of 15 females kept on normal food or subjected to starvation for 24 h. Q-RT-PCR was essentially performed following recently described methods^46^. The following primers were used as follows: *Rpl32* forward (5′-CCG CTT CAA GGG ACA GTA TC-3′), *Rpl32* reverse (5′-GAC AAT CTC CTT GCG CTT CT-3′); *Dilp2* forward (5′-CTG AGT ATG GTG TGC GAG GA-3′), *Dilp2* reverse (5′-ACAAACTGCAGGGGATTGAG-3′); *Dilp3* forward (5′-GGCCGCAAACTGCCCGAAAC-3′), *Dilp3* reverse (5′-GGGAACGGTCTTCGAAGCCATCG-3′); *Dilp5* forward (5′-ATGCTGAGGGTTGCCTGTCCC-3′), *Dilp5* reverse (5′-TCCAAATCCGCCAAGTGGTCCTC-3′).

### Statistical analyses

All statistical analyses were performed using GraphPad Prism Software (version 5). Life span and starvation survivorship were analysed by log-rank assays. Other parameters were evaluated using the unpaired two-tailed Student’s *t*-test. All data are presented as mean values ± SEM.

## Additional Information

**How to cite this article**: Li, Y. *et al.* Octopamine controls starvation resistance, life span and metabolic traits in *Drosophila. Sci. Rep.* **6**, 35359; doi: 10.1038/srep35359 (2016).
